# Tinnitus treatment with acamprosate: double-blind study

**DOI:** 10.1016/S1808-8694(15)31266-0

**Published:** 2015-10-20

**Authors:** Andréia A. Azevedo, Ricardo R. Figueiredo

**Affiliations:** 1 Otorhinolaryngologist, OTOSUL – Otorrinolaringologia Sul-fluminense, Hospital São Camilo, Volta Redonda, RJ.; 2 Otorhinolaryngologist, OTOSUL – Otorrinolaringologia Sul-fluminense, Hospital São Camilo, Volta Redonda, RJ.

**Keywords:** tinnitus, acamprosate

## Abstract

Nowadays, the treatment of tinnitus is still a great challenge for the otolaryngologists. Many facts remain unknown in its pathophysiology, leading to many different therapies, with irregular results. Acamprosate is a drug used in alcoholism treatment, due to its regulating effects in glutamatergic and GABA neurotransmission, and has never been used before in the treatment of tinnitus **Aim**: To evaluate efficacy and safety of the acamprosate in the treatment of sensorineural tinnitus. **Study design:** randomized clinical trial. **Material and Method**: 50 patients with sensorineural tinnitus were divided into two groups: 25 received acamprosate and 25 placebo, for a period of 3 months, in a prospective double-blind study, being analyzed for its efficacy and safety by the subjective score from 1 to 10 given by the patient. **Results**: We found a high index of success in the relief of tinnitus, about 86.9%. In 47.8% of the cases we found more than 50% relief. The incidence of side effects was low, 12%, all of them mild. **Conclusion**: Acamprosate, a drug used in the treatment of alcoholism, is a safe and successful alternative for sensorineural tinnitus’ treatment.

## INTRODUCTION

Many different types of tinnitus treatment have recently emerged, with partially satisfactory results, reflecting the incomplete understanding that people have about pathophysiology ^1^. To many, tinnitus is a continuous problem to Otorhinolaryngologists ^2^, with poor and frustrating clinical results and dissatisfied patients. In view of this situation, any therapeutic option that may add new possibilities of improvement should at least be considered for our current therapeutic arsenal.

Among the many theories already created for tinnitus pathophysiology, one of the most widely accepted today is the “excitotoxicity”, based on excessive release of excitatory neurotransmitter glutamate in the peripheral and central auditory pathways, which results in overexpression of synaptic receptors type NMDA (N-Methyl D-Aspartate), causing edema and rupture of primary auditory neurons, owing to excessive input of calcium into the cells. Owing to excessive NMDA receptors in the neurons, they get more sensitive to excitotoxicity of the glutamate, leading to a vicious cycle disseminated to the whole auditory pathways (epilepsy of the auditory pathways)^1^, ^3^.

Glutamate, the main excitatory neurotransmitter amino acid of the auditory system, both peripheral and central, is formed from alpha-oxo-glutamate, intermediate in Krebs cycle, by the action of GABA (Gamma-Amino-Butiric Acid)-aminotransferase^1^, ^4^.

**Chart 1:**
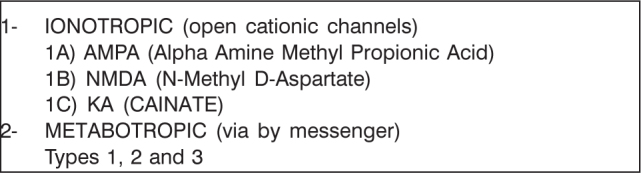
Glutamatergic receptors.

Synaptic receptors for glutamate may be divided as follows (Chart 1)^3^, ^5^:

AMPA is the English acronym for alpha-amino-3 hydroxi-5 methyl-isoxazol-propionic acid. It has fast excitatory responses and in most physiological situations, it is relatively impermeable to calcium ^1^. These are the main involved cells with physiological synaptic transmission of auditory pathways and it is believed that they are present in all or almost all neurons of the central nervous system^3^, ^5^.

AMPA receptors may present with a wide range of subunits, with wide structural variations, including at the level of post-transcriptional affections (RNA-m), which may result in different functional properties^5^. In auditory pathways, especially, there are variants of the AMPA receptors with high permeability to calcium ^3^.

NMDA is an English acronym to N-methyl-D-aspartate. It has slower excitatory responses, is highly permeable to calcium and blocked by magnesium ^1^. It does not participate in the physiological neurotransmission of auditory pathways, and it is overexpressed in pathological conditions. However, they seem to be involved in some neuroplasticity mechanisms ^5^.

The role of kainite receptors in neurotransmission of auditory pathways is still not very clear. In the central nervous system, they seem to perform a secondary role in physiological neurotransmission and in some cases, they may also be harmful because of excessive permeability to calcium. Some studies in rats suggested its presence at the level of the lateral superior olivary complex 3, 5.

In metabotropic receptors, transmission of impulse is triggered by second intracellular messengers ^1^.

The role of neurotransmission in auditory pathways has still not been completely understood. Some subtypes present regulation action of glutamatergic transmission, others maximize it, in situations of excessive stimulus (noise, for example)^5^, ^6^.

Efferent auditory system has regulating function. Information coming from the auditory cortex is condensed and organized in lateral and medial olivary complexes and then it is sent to the cochlea, through medial and lateral olivary cochlear tracts (TOM and TOL, respectively).

TOM is responsible for efferent innervation of outer hair cells (OHC), with fibers coming from homolateral and contralateral medial olivary complexes. The main neurotransmitters are acetylcholine and GABA, the latter specially in the apical regions. TOL is responsible for efferent innervation of inner hair cells (IHC), and it has only homolateral fibers. The main neurotransmitters are acetylcholine, GABA and dopamine 1, 3.

Tinnitus may be classified in different ways. In our opinion, the most appropriate way is the one that divides them into para-auditory tinnitus (muscle and vascular) and auditory tinnitus (generated by external, middle or inner ears, as well as the peripheral and central pathways). The tinnitus originated from inner ear or central and peripheral pathway damage is named sensorineural 1, 3.

Among the therapeutic possibilities for sensorineural tinnitus, we can include drugs (such as ginkgo biloba Egb 761, clonazepam, carbamazepine, piribedil), zinc replacement (in elderly and in cases of hypozincemia), electro-stimulation, bio-feedback and habituation therapies (such as T.R.T. - tinnitus retraining therapy).^1^, ^4^, ^5^ According to some authors, average efficacy of therapeutic success with drugs ranges at about 50%^7^.

The chemical name acamprosate is calcium acetylhomotaurinate or acetylaminopropanosulfonate whose chemical structure is analogous to some pharmacologically active amino acids, such as GABA, glutamate and taurine.

Acamprosate acts both on the glutamatergic system (excitatory) and in the inhibitory GABA system, counting on the dual mechanism to treat tinnitus. No other drug used in the treatment of tinnitus has a concomitant action in both systems 8, 9.

Acamprosate increases the number of reuptake sites of GABA and modified GABA reuptake in rats, with global effects of increased GABA transmission, which inhibits the excitation of auditory pathways ^8^.

Acamprosate reduces the effect of excitatory amino acids (glutamate) in the central nervous system, especially its excitatory action over NMDA receptors, probably owing to an action at the level of calcium channel blockers 8, 9.

We intended to assess the efficacy and safety of acamprosate in the treatment of tinnitus of sensorineural origin by performing a double-blind randomized study.

## MATERIAL AND METHOD

We selected 50 patients with tinnitus seen at OTOSUL, Otorrinolaringologia Sul-fluminense, Hospital São Camilo, Volta Redonda, RJ, between October 2003 and October 2004. The inclusion criteria were tinnitus of sensorineural etiology, excluding the cases of external and middle ear disease and concomitant temporomandibular joint disorders. Pure tone audiometry, vocal audiometry and immittanciometry were performed in all patients, and we excluded those with conductive and mixed hearing loss and those with tympanogram curve types A-r, A-d, C and B. The audiometer used was AMPLAID A 177 PLUS, and the Immittanciometer was AMPLAID 750. We considered the thresholds up to 25 dB (normal hearing), 26 to 40 dB (mild loss), 41 to 70 dB (moderate loss), 71 to 90 dB (severe loss) and over 90 dB (profound loss).

The patients were classified according to the following parameters:

• age
• gender
• continuous or intermittent tinnitus
• uni or bilateral tinnitus
• tinnitus characteristics (type of associated noise)
• time of tinnitus
• associated symptoms (hypo and hyper loss, dizziness, ear fullness)
• probable tinnitus etiology
• previous use of drugs to treat tinnitus
• baseline tinnitus score, given by the patient, ranging from 0 to 10, according to how much it disturbed the patient

Patients were asked to score tinnitus from 1 to 10 according to how much they were disturbed by it. We allowed 0.5 scores as well.

Patients were randomly divided into 2 groups of 25 patients: one group received acamprosate 333mg, TID, and the other received placebo, TID, both groups for 90 days. All details of the study were clarified to the patients by the assistant physician and they all signed the informed consent term. It was a double-blind study approved by the Medical Ethical Committee, Hospital São Camilo.

Patients were analyzed at days 30, 60 and 90 after use of the drug and at each visit, the patient scored the tinnitus status, as well as reported side effects.

The statistical analysis was performed based on the following methods:

• to compare quantitative data between the two groups (acamprosate and placebo) we used t Student test for independent samples or the Mann-Whitney test;
• to compare qualitative data we applied the chi-square test (χ^2^) or Fisher exact test;
• to analyze the progression of tinnitus scale (quantitative data) throughout time (four assessments within 3 months) with the treatment with acamprosate and placebo we performed Friedman Variance Analysis. The test of multiple comparisons based on the statistics of Friedman was applied to identify which moments were different. The test of multiple comparisons is a test complementary to Variance Analysis.

The criterion for determination of significance was adopted as 5%, that is, when p value of the statistical test was smaller or equal to 0.05, meaning there was statistical significance. The statistical analysis was processed by the statistical software SAS System.

## RESULTS

The age of patients ranged from 35 to 82 years (mean age of 60 years, median of 60.5 years). The duration of symptoms in months ranged from 1 to 420 (mean of 101.78 months, median of 60 months).

We observed that 58% of the patients were male and 42% were female patients. The type of tinnitus most frequently reported was wheezing sound (62% of the patients) and whistle (46% of the patients). They also reported sounds such as engine, pressure-cooking pain, and cicada, and 16% of the patients had more than one type of tinnitus concomitantly.

As to time of symptomatology (tinnitus), we observed that 9.76% had recent tinnitus (less than 1 year), 53.65% had intermediate tinnitus (between 1 and 7 years) and 36.59% had old cases of tinnitus (for more than 7 years).

It was observed that 58% of the patients presented bilateral tinnitus, 72% had continuous tinnitus and 64% reported associated symptoms. Out of the total, 59.4% reported hearing loss, 46.9% reported dizziness, 15% had ear fullness and 9.3% had hyperacusis. Moreover, 52% of the patients had already taken some type of drug to treat tinnitus.

**Graph 1:**
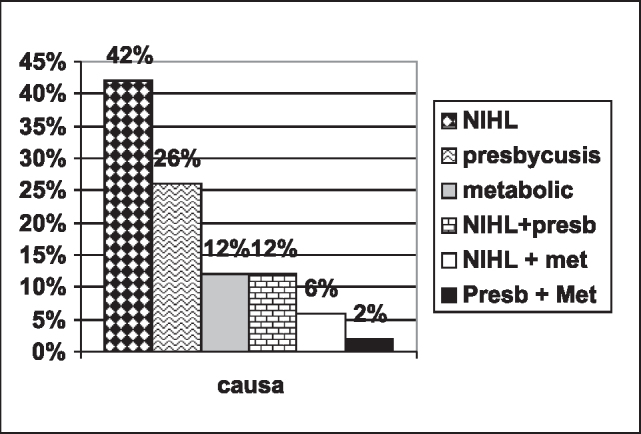
Probable tinnitus etiology.

As to probable etiology of tinnitus, the distribution is shown in Graph 1.

Exposure to noise as probable etiological factor occurred in 60% of the cases. Twenty percent of the patients presented a probable multifactorial etiology for tinnitus.

**Graph 2:**
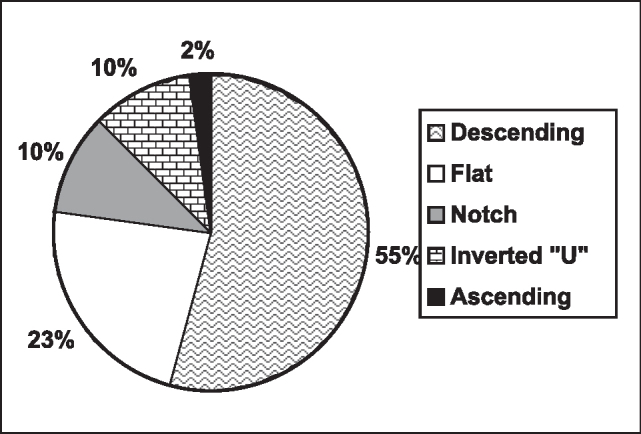
Type of audiometric curve found.

As to auditory thresholds, we observed normal audiogram in 2 patients (4%), mild hearing loss in 30 (60%), moderate in 10 (20%), severe in 6 (12%), and profound in 2 (4%). Out of the patients with threshold abnormalities, the type of audiometric curve found was distributed as shown below in Graph 2.

Considering the 50 patients, 9 interrupted treatment before the end of assessment, 2 in the acamprosate group and 7 in the placebo group. One patient (50%) of the acamprosate group and 5 (71.4%) in the place group suspended treatment owing to side effects. The other 3 patients out of 50 suspended treatment owing to family pressure.

The proportion of improvement at day 90 (any improvement in score, that is, the percentage of improvement different from zero) in the group treated with acamprosate (86.9%) was significantly greater (p=0.004) than in the group treated with placebo (44.4%).

The proportion of improvement at 90 days equal or greater than 50% in the group treated with acamprosate (47.8%) was significantly (p=0.012) higher than in the group treated with placebo (11.1%).

In the group treated with acamprosate we did not observe worsening in score. Three patients (13.04%) did not report improvement, 9 (39.13%) reported improvement below 50% and 11 patients (47.83%) reported improvement higher than 50%. Three subjects (13.04%) reported that tinnitus had disappeared.

To assess the progression of tinnitus score throughout time, separately for each group, we performed Friedman Variance Analysis. This analysis checks whether there is significant variation (fall or increase) throughout time. The tests of multiple comparisons based on Friedman statistics were applied to identify which times differed.

Tables 1 and 2 show mean, standard deviation (SD), median, minimum and maximum of the scale at four moments and the corresponding level of significance (p value) of Friedman for the group treated with acamprosate and placebo, respectively. The moments that differed, identified by the test of multiple comparisons, were highlighted in the column of significant difference, at 5% level.

**Table 1:** Longitudinal analysis of tinnitus scale for the group with Acamprosate (Note: SD= standard deviation; n= total number of patients that concluded 90 days of treatment; mean, median, minimum and maximum in relation to the score.)

Tinnitus scale	n	Mean	SD	Median	Minimum	Maximum	p value	significant differences
Baseline	23	6,74	2,53	7	3	10	0,0001	Compared to 30, 60 and 90d
Score – 30d	23	5,39	2,29	5	2	10		30d, compared to 60 e 90d
Score – 60d	23	3,98	2,62	4	0	9		60d, compared to 90d
Score – 90d	23	2,87	2,70	2	0	10

**Table 2:** Longitudinal analysis of tinnitus scale for the Placebo group (Note: SD= standard deviation; n= total number of patients that concluded 90 days of treatment; mean, median, minimum and maximum in relation to the score.)

Tinnitus scale	n	Mean	SD	Median	Minimum	Maximum	p value	significant differences
Baseline	18	5,72	2,47	6	2	9	0,22	No differences
Score – 30d	18	5,51	2,33	5,5	1,5	9		
Score – 60d	18	5,27	2,48	5,5	1	9		
Score – 90d	18	5,17	2,77	4,5	1	10		

**Graph 3:**
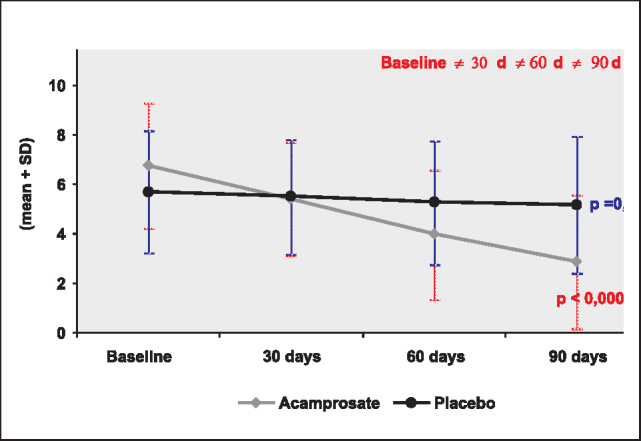
Tinnitus scale according to the group after 3 months of treatment.

Based on Analysis of Variance, we observed that there was significant decrease in tinnitus scale (p<0.0001) in the group with acamprosate as a result of time. We observed in the column of significant differences that there was significant decrease from baseline to the other situations of assessment (30 days) and the 3rd and 4th assessments (60 and 90 days), as shown in Graph 3.

The Analysis of Variance showed that there was no significant variance in tinnitus scale (p=0.22) in the group with placebo throughout the 90 days of treatment, as shown in Graph 3.

There was no statistically significant difference in results of the different groups concerning age, gender, etiology, time and type of tinnitus, level of hearing loss and type of audiometric curve, both in the acamprosate and placebo groups.

We observed that in the group with acamprosate there was significant decrease in the tinnitus scale after the first month of treatment, whereas in the placebo group there was no significant variation during the three months.

As to side effects, analyzed by Fisher exact test, we did not observe statistically significant difference (p=0.35) between the groups taking acamprosate (12%) and placebo (20%). The observed side effects with acamprosate were mild (epigastralgia, choking), leading to interruption of treatment in 1 case, in which the patient developed depression, whose association with acamprosate, even though theoretically possible, is questionable in our opinion.

## DISCUSSION

Tinnitus is a very frequent symptom that affects, based on many studies, from 14 to 32% of the general population^1^, ^13^. In 20% of the cases, tinnitus had some repercussion in the life of the patients, which may be event disabling in about 5% of the cases 1, 13.

Treatment of tinnitus is very difficult both for the physician and the patient, resulting in irregular outcomes, regardless of drug or clinical approach. Some non-medication alternatives, such as habituation (Tinnitus Retraining Therapy, T.R.T.) may be quite effective 10, 11, but the results are perceived in the long run (18 to 24 months), requiring complete integration of the patients and constant meetings with groups of patients ^23^. Considering such situation, any new perspective seems to be received as a precious new alternative.

Studies carried out with ginkgo biloba Egb 761 extract, at dosage of 120mg BID showed improvement of tinnitus in 57.5% of the cases, better than the improvement obtained with 80 mg BID (42.5%)^12^.

Treatment of tinnitus with carbamazepine, in progressively increasing doses of 50 to 600 mg a day, defined by positive lidocaine test, may result in improvement of 50% of the cases of tinnitus^14^.

Other drugs already studied for treatment of tinnitus are baclofen (improvement in about 9.7%)^15^, caroverine (63.3%)^16^, piribedil (92.6%) ^17^, nimodipine (16.13%)^18^, clonazepam (32%)^19^ and trimetazidine (89%)^20^, ^21^. Only the studies that investigated baclofen and caroverine were double blind placebo-controlled studies. Trimetazidine, antiischemic drug used in Europe to treat tinnitus and vertigo, is more effective in recent tinnitus (less than 1 year of duration)^21^.

In a study performed by Murai et al.^22^ in 1992, the authors retrospectively assessed the use of many different drugs in the treatment of tinnitus, including clonazepam and flunarizine and they found rates of symptom improvement ranging from 33 to 56%.

We did not find in the literature any reference to use of acamprosate in the treatment of tinnitus. Its dual action mechanism, which reduces the glutamatergic transmission (excitatory) and increases the GABA activity (inhibitory), together with its excellent tolerability, makes it an excellent and promising drug for tinnitus.

Even though the subjective assessment by scores may differ between different patients (given that tinnitus of the same intensity may bother patients differently), we intended to assess the therapeutic result of the medication, and improvement in the score was the most significant factor. In our study, the percentage of tinnitus improvement was significant (86.9%) and in 47.8% of the cases the improvement was greater than 50%. We observed decrease in the score given to the tinnitus in the acamprosate group as the 90 days went by. The improvement rate was greater than that of other drugs, and in the case of trimetazidine (89%) and piribedil (92.6%) data were not collected from double-blind placebo controlled studies. The study with piribedil was carried out by general clinicians, who concomitantly assessed other symptoms, such as vertigo and memory deficit^17^. It is important to point out that there was no statistically significant difference in improvement comparing factors such as age, gender, etiology, type and duration of tinnitus, as well as severity of hearing loss and type of audiometric curve, opening a perspective of treatment for all cases of tinnitus of sensorineural cause.

Future studies should determine the effects of acamprosate in relieving tinnitus for periods longer than 90 days.

## CONCLUSION

In the little understood universe of tinnitus, of multifactorial etiology and treatment with variable results, all forms of treatment that may generate some relieve to patients should be considered as a therapeutic option. For this reason, acamprosate, a drug used for the treatment of alcohol abuse, with dual action mechanism at the inner ear and auditory pathways and excellent tolerability, should be part of our therapeutic arsenal.
